# An accumulated mutation gained in mosquito cells enhances Zika virus virulence and fitness in mice

**DOI:** 10.1128/jvi.01251-24

**Published:** 2024-10-16

**Authors:** Xiao-Xuan Fan, Rui-Ting Li, Yi-Bin Zhu, Qi Chen, Xiao-Feng Li, Tian-Shu Cao, Hui Zhao, Gong Cheng, Cheng-Feng Qin

**Affiliations:** 1 State Key Laboratory of Pathogen and Biosecurity, Academy of Military Medical Sciences, Beijing, China; 2 New Cornerstone Science Laboratory, Tsinghua University-Peking University Joint Center for Life Sciences, School of Basic Medical Sciences, Tsinghua University, Beijing, China; 3 Institute of Infectious Diseases, Shenzhen Bay Laboratory, Shenzhen, China; 4 Research Unit of Discovery and Tracing of Natural Focus Diseases, Chinese Academy of Medical Sciences, Beijing, China; University of North Carolina at Chapel Hill, Chapel Hill, North Carolina, USA

**Keywords:** Zika virus, accumulated mutation, mosquito, mammal, virulence, fitness

## Abstract

Zika virus (ZIKV) remains a significant public health threat worldwide. A number of adaptive mutations have accumulated within the genome of ZIKV during global transmission, some of which have been linked to specific phenotypes. ZIKV maintains an alternating cycle of replication between mosquitoes and vertebrate hosts, but the role of mosquito-specific adaptive mutations in ZIKV has not been well investigated. In this study, we demonstrated that serial passaging of ZIKV in mosquito Aag2 cells led to the emergence of critical amino acid substitutions, including A94V in the prM protein and V153D and H401Y in the E protein. Further characterization via reverse genetics revealed that the H401Y substitution in the E protein did not augment viral replication in mosquitoes but significantly enhanced neurovirulence and lethality compared with those of the wild-type (WT) virus in mice. More importantly, the H401Y mutant maintained its virulence phenotype in mice after propagation in mosquitoes in mosquito-mouse cycle model. In particular, recombinant ZIKV harboring the H401Y substitution showed enhanced competitive fitness over WT ZIKV in various mammalian cells and mouse brains, but not in mosquito cells. Notably, the H401Y substitution in the ZIKV E protein has been detected in recent isolates derived from both mosquitoes and humans in Asia and the Americas. In summary, our findings not only identify a novel virulence determinant of ZIKV but also highlight the complexity of the relationship between the evolution of vector-borne viruses and their clinical outcome in nature.

**IMPORTANCE:**

Zika virus (ZIKV) is an important arbovirus with a global impact. Experimental evolution by serial passaging of ZIKV in susceptible cells has led to the identification of a panel of critical amino acid substitutions with specific functions. Herein, we identified a mosquito cell-derived substitution, H401Y, in the ZIKV E protein via experimental evolution. The H401Y substitution significantly enhanced viral virulence and fitness in mammal cells and mice. Notably, the H401Y substitution has been detected in recent mosquito and human isolates from regions spanning Asia to the Americas. Our work elucidates unrecognized virulence determinant in the ZIKV genome that warrants urgent attention. Moreover, the findings underscore the critical need for extensive molecular surveillance and rigorous clinical observation to establish the potential impact in natural circulation. These endeavors are crucial for unraveling the potential of mutation to act as a catalyst for future epidemics, thereby preempting the public health challenges it may pose.

## INTRODUCTION

Zika virus (ZIKV), a member of the mosquito-borne orthoflavivirus, maintains an alternating cycle of replication between mosquitoes and vertebrate hosts (1, 2). The widespread transmission of ZIKV is a public health issue of global concern. Between 2015 and 2017, the introduction of ZIKV into the Western Hemisphere extended the endemic range of the virus to over 80 countries, resulting in millions of infections (3). ZIKV infection is associated with not only mild symptoms such as fever, arthralgia, myalgia, and conjunctival congestion but also severe complications like Guillain-Barré syndrome and congenital Zika syndrome in newborns (4, 5). No vaccines or antiviral agents against ZIKV have been commercially licensed.

ZIKV belongs to the *Orthoflavivirus* genus within the *Flaviviridae* family (6) and is an enveloped, positive-sense single-stranded RNA virus with a genome approximately 11 kb in length. The genome comprises 5′ and 3′ untranslated regions (UTRs) flanking an open reading frame (7). Several evolutionarily conserved cis-acting RNA elements are located in 5′ and 3′ UTRs and are involved mainly in genome cyclization, replication, and translation (8). The coding sequence is translated into a polyprotein encompassing all the viral proteins: three structural proteins [capsid (C), premembrane (prM) or membrane (M), and envelope (E)] and seven nonstructural proteins (NS1, NS2A, NS2B, NS3, NS4A, NS4B, and NS5). The encoded polyprotein precursor is further cleaved co and post translationally by viral and host proteases (9). Orthoflaviviruses, which lack proofreading functions, present high mutation rates, leading to various adaptive mutations in response to environmental pressures (10, 11).

A panel of critical mutations has emerged during the global transmission of ZIKV (12–20). We previously demonstrated that the amino acid substitution prM-S17N (S139N) enhances ZIKV neurovirulence (13), and that NS1-A188V and C-T106A promote viral transmission (14–16). Furthermore, the neurovirulence determinants prM-E21K and C-K101R are associated with the virulence of the African lineage (17, 18). Additionally, Shan et al. have demonstrated that the E-V473M substitution enhances viral virulence and transmission (19). Notably, Liu et al. experimentally demonstrated that four amino acid substitutions that preceded introduction into the South Pacific and the Americas increased ZIKV infection and transmission efficiency by mosquitoes (21). These findings highlight the potential for more virulent ZIKV strains to emerge in the future.

Laboratory evolutionary studies involving *in vitro* and *in vivo* serial passaging of ZIKV in susceptible cells or hosts have identified various adaptive mutations with specific functions (22–24). During passage in neonatal mice, Guo et al. obtained an adaptive ZIKV strain with two amino acid substitutions (E143K and R3394K), enhancing the neurovirulence of ZIKV in newborn mice (22). Liu et al. serially passaged ZIKV in neonatal mice and discovered a single D67N substitution in the E protein that markedly increased virulence and neurotropism (23). More importantly, Regla-Nava et al. identified a novel substitution, I39V, leading to enhanced transmission and infectivity, via serial passaging of ZIKV in a mosquito-mouse transmission model (24).

In this study, via serial passaging of ZIKV in mosquito Aag2 cells, we identified a unique mosquito-derived amino acid substitution, H401Y, in the E protein. Surprisingly, this substitution did not alter viral fitness within the mosquito host but led to a significant increase in infectivity, virulence, and competitive fitness in mammals.

## RESULTS

### Accumulated ZIKV mutations through serial passaging in mosquito cells

**Fig 1:**
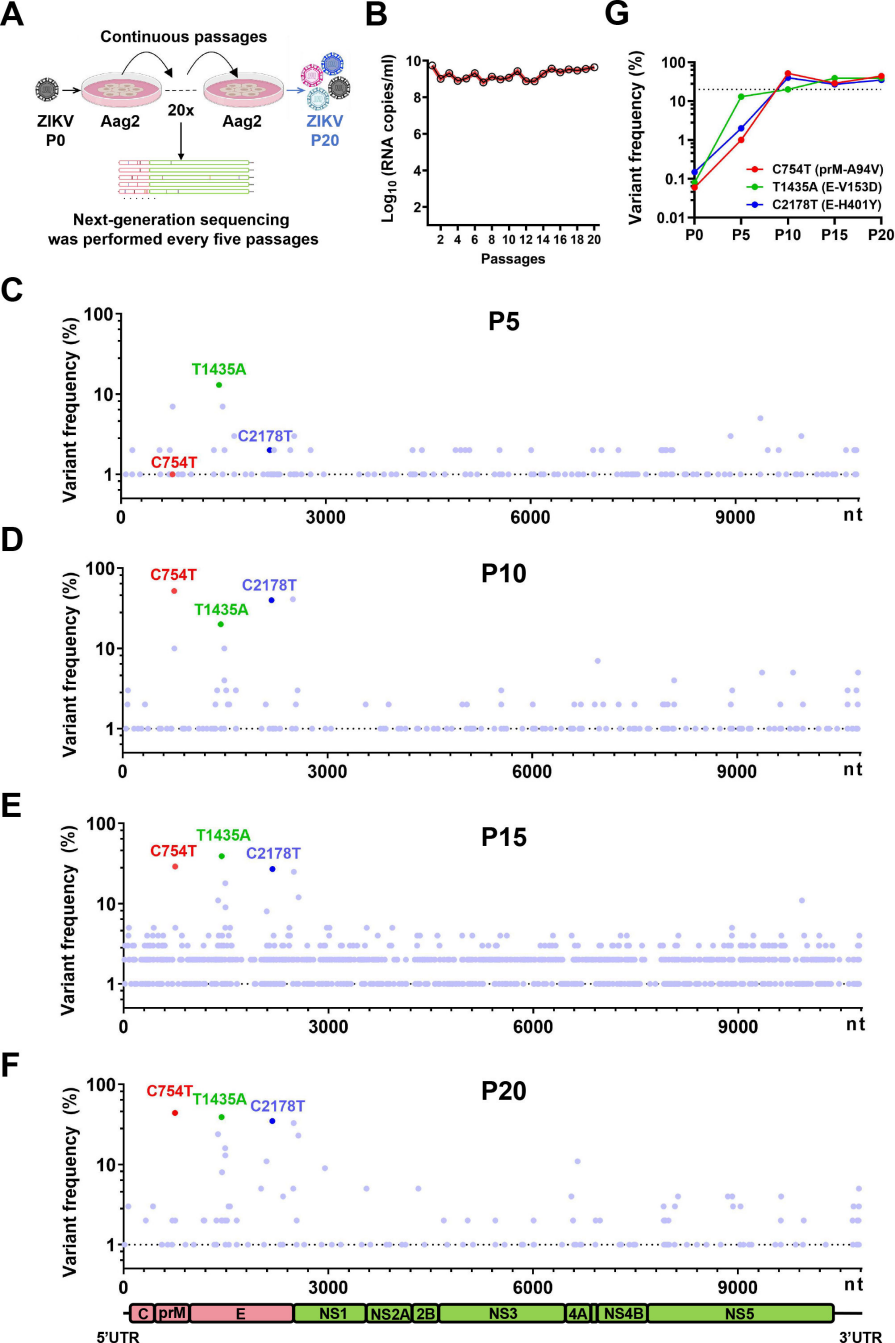
Accumulation of mutations within the ZIKV genome during continuous passaging in Aag2 cells. (**A**) Experimental scheme. The ZIKV strain FSS13025 was cultured through 20 serial passages in Aag2 cells. Next-generation sequencing was employed every five passages to characterize the mutation of the viral genome. (**B**) The number of viral RNA copies was quantified via RT-qPCR. (**C–F**) Mutation landscape at the 5th, 10th, 15th, and 20th passages. Key mutations with frequencies ≥1% are indicated, with the C754T, T1435A, and C2178T mutations having the highest frequencies of occurrence. The dotted line represents a 1% mutation frequency. The C754T, T1435A, and C2178T mutations are colored in red, green, and blue, respectively. (**G**) Mutation frequency tracking of C754T, T1435A, and C2178T at P0, P5, P10, and P20. The dotted line represents a 20% mutation frequency.

To obtain mosquito-accumulated mutations, the ZIKV strain FSS13025 was serially passaged 20 times in Aag2 cells, followed by next-generation sequencing at predefined intervals (Fig. 1A). Despite minor fluctuations, virus replication in Aag2 cells remained relatively stable throughout the passages (Fig. 1B). By the 20th passage, 191 mutation sites exhibiting a mutation frequency of at least 1% were identified across the whole genome (Fig. 1C through F), with 24 sites surpassing a 3% mutation frequency. Six of these mutations were synonymous, 17 were nonsynonymous mutations, and 1 was in the 3′ UTR. Notably, C754T, T1435A, and C2178T displayed higher frequencies of occurrence (44%, 39%, and 35%), leading to the amino acid substitutions A94V in the prM protein and V153D and H401Y in the E protein, respectively. The variant frequencies of three mutations in the starting stock virus were less than 1%, increased to over 20% after the tenth passage, and became stable until the 20th passage (Fig. 1G). These three mutations, which are putatively beneficial for mosquito adaptation, were selected for further analysis.

### Construction and characterization of recombinant ZIKV with mosquito accumulated mutations

**Fig 2:**
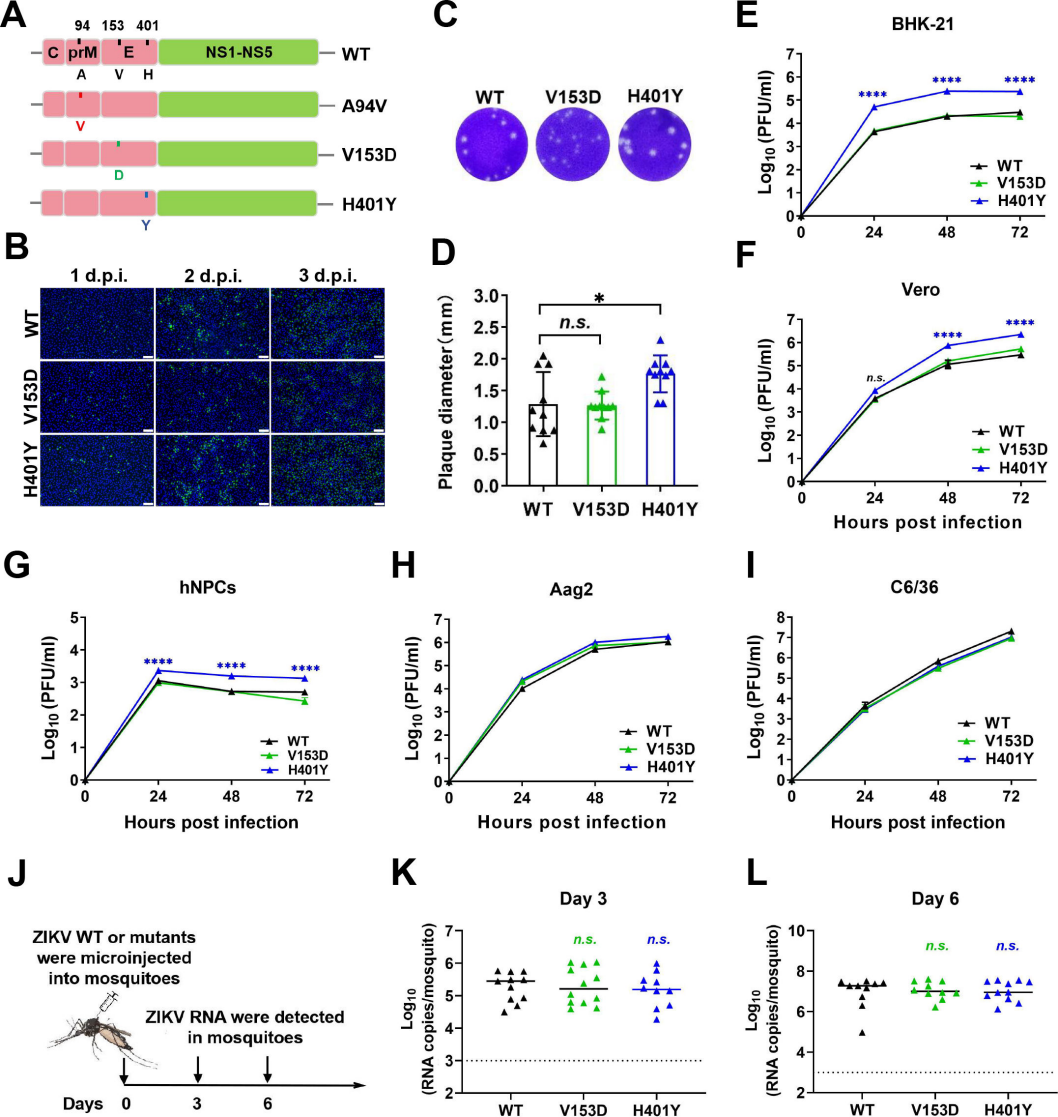
Construction and characterization of recombinant ZIKV with selected substitutions. (**A**) Schematic diagram of WT and mutant ZIKVs. A94V, V153D, and H401Y are denoted by red, green, and blue lines, respectively. (**B**) Results of the indirect immunofluorescence assay (IFA) of WT, H401Y, and V153D infected BHK-21 cells at an MOI of 0.1. Viral protein expression was detected with a ZIKV-E-specific mAb at the indicated time points. Scale bar, 100 µm. (**C and D**) Plaque morphology (**C**) and plaque sizes (**D**) of WT, H401Y and V153D in BHK-21 cells (*n* = 10). Statistical significance was determined using one-way ANOVA (*P < 0.05). (**E–H**) Growth curves of WT, H401Y, and V153D in BHK-21 (**E**), Vero (**F**), hNPC (**G**), Aag2 (**H**), and C6/36 (**I**) cells. The supernatants were harvested at the indicated time points and subjected to PFU quantification. Two-way ANOVA was performed for statistical analysis (*P < 0.05, ***P < 0.001, ****P < 0.0001). (**J**) Mosquito infectivity assessment of recombinant ZIKV. (**K and L**) Infectivity of ZIKV mutants in mosquito hemocoel tissues. Mosquitoes were collected at 3 days (**K**) or 6 days (**L**) after the microinjection for infection levels via RT-qPCR, and the *P* values were determined using one-way ANOVA. The data are shown as means ± SEM. The dotted line represents the detection threshold.

To investigate the biological functions of these accumulated mutations acquired in Aag2 cells, A94V, V153D, and H401Y substitutions were engineered into an infectious clone of the ZIKV strain FSS13025 (WT) (Fig. 2A). The A94V mutant was nonviable in BHK-21 cells; hence, subsequent assays focused on the V153D and H401Y mutants. An indirect immunofluorescence assay (IFA) revealed that both the V153D and H401Y mutants efficiently replicated in BHK-21 cells (Fig. 2B). Additionally, a plaque-forming unit (PFU) assay revealed that compared with both the WT and V153D mutant virus, the H401Y mutant virus produced larger plaques in BHK-21 cells (Fig. 2C and D). Consistently, growth curve analysis across a range of ZIKV-susceptible mammalian cell lines, including BHK-21, Vero, and human neural progenitor cells (hNPCs), indicated that H401Y exhibited enhanced replication (Fig. 2E through G). However, they did not affect viral replication in Aag2 cells or C6/36 cells (Fig. 2H through I). Interestingly, despite their identification in mosquito cells, *in vivo* studies involving intrathoracic injection into female *Aedes aegypti* (*Ae. aegypti*) mosquitoes revealed comparable replication kinetics for both V153D and H401Y mutants at 3 and 6 days post infection (d.p.i.) (Fig. 2J through L). Collectively, these findings underscore the role of the H401Y substitution in increasing ZIKV replication in mammalian cells without conferring a replication advantage in mosquito hosts.

### The H401Y substitution augments the neurovirulence and lethality of ZIKV in mice

**Fig 3:**
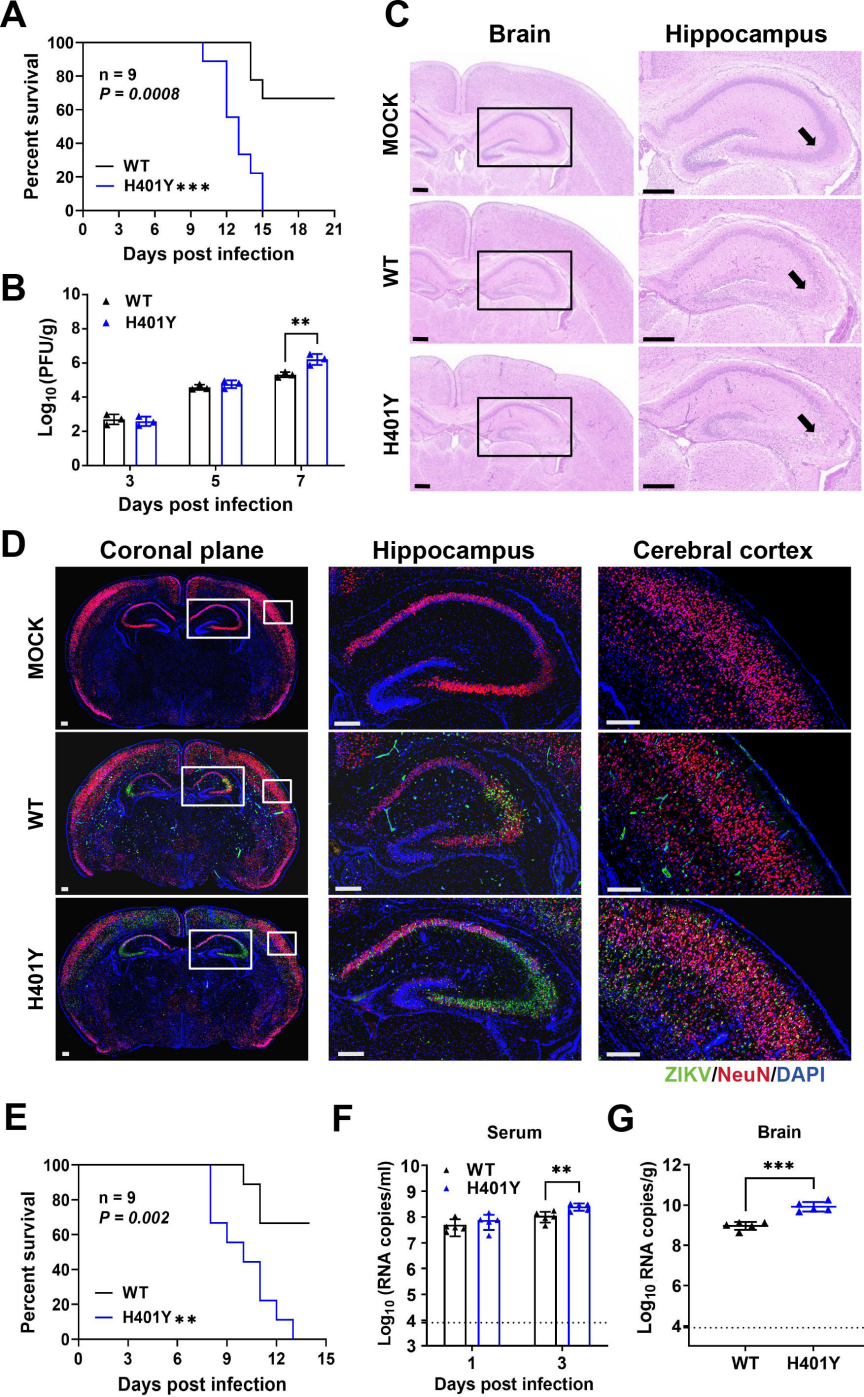
Neurovirulence and neuroinvasiveness of the H401Y mutant virus in mice. (**A**) Neurovirulence in neonatal mice. Groups of one-day-old CD-1 mice (*n* = 9) were inoculated intracranially with 10 PFU of WT or H401Y. Survival curves were analyzed by Log rank test (***P < 0.001). (**B**) Viral loads in mouse brains were detected at the indicated times via PFU assay (*n* = 3). Two-way ANOVA was performed for statistical analysis (***P < 0.001). (**C**) Representative histopathological images of the coronal planes of mouse brain tissues at 9 d.p.i.; scale bar, 300 µm. (**D**) Representative immunofluorescence images of coronal plane of WT- or H401Y-infected brains dissected at 9 d.p.i. with NeuN (red) and ZIKV (green) antibodies; Scale bar, 200 µm. (**E**) Neuroinvasiveness in mice. A group of three-week-old A129 mice (*n* = 9) were intraperitoneally inoculated with 100,000 PFU of WT or H401Y virus. Log rank test was performed for statistical analysis (**P < 0.01). (**F**) Viremia was measured using RT-qPCR. One-way ANOVA was performed for statistical analysis (****P < 0.0001). (**G**) Viral loads in the brain tissues of the infected mice at 7 d.p.i. (*n* = 5). The value represent mean ± SD. Unpaired *t* test was performed for statistical analysis (**P < 0.01; ***P < 0.001; *P* > 0.05, n.s.). (**F and G**) The dotted line represents the detection threshold.

To assess the *in vivo* impact of the H401Y substitution, we next analyzed its neurovirulence phenotype in an established suckling mouse model (14, 18, 19). Compared with WT virus, one-day-old CD-1 mice intracranially injected with 10 PFU of the H401Y mutant virus presented more severe symptoms, earlier death, and increased mortality (Fig. 3A). The average survival time (AST) of H401Y-inoculated mice was significantly reduced (12.9 vs 18.8). The virus loads in the brains of H401Y-infected mice were approximately 10-fold greater than those in the brains of mice infected with WT viruses at 7 d.p.i., both in terms of infectious particle and viral RNA abundance (Fig. 3B). Histopathological evaluations of brain tissue showed that more extensive damage in hippocampal structures following H401Y infection than following WT virus infection (Fig. 3C). Immunofluorescence staining corroborated these findings, revealing a higher proportion of viral antigen-positive cells, especially within the hippocampus, in H401Y-infected mice (Fig. 3D). These data collectively confirm that the ZIKV variant harboring the H401Y substitution exhibits elevated neurovirulence in neonatal mice.

Comparative investigations of neuroinvasive properties were next conducted using an A129 mouse model (16, 25). Three-week-old A129 mice that were intraperitoneally injected with 100,000 PFU of the H401Y or WT virus presented distinct outcomes. Mice infected with H401Y viruses died earlier, with all the mice perishing by 13 d.p.i. In contrast, mouse mortality was only 33% in the WT group over a 14-day window (Fig. 3E). The H401Y substitution led to more profound viremia and higher viral RNA abundance in brain tissue (Fig. 3F and G). Together, these results demonstrate that the H401Y substitution intensifies the lethality of ZIKV in A129 mice.

### The H401Y substitution enhances the competitive fitness of ZIKV in mammalian cells and mouse brains

**Fig 4:**
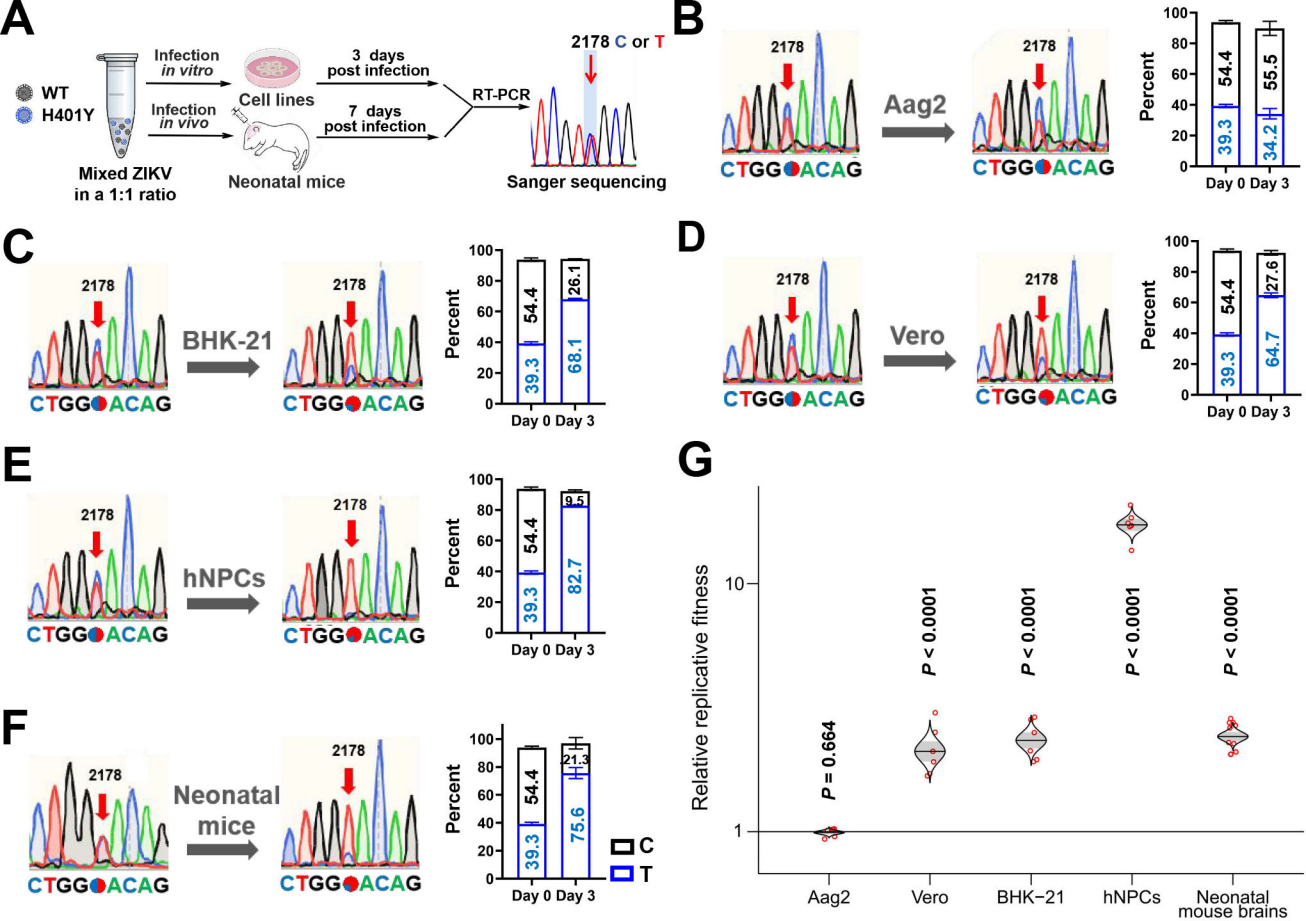
*In vitro* and *in vivo* fitness comparison of WT and H401Y mutants. (**A**) Schematic representation of the study design. WT and H401Y strains were mixed at a 1:1 PFU ratio and used to infect Aag2, Vero, BHK-21, hNPCs, and neonatal mice, respectively. Viral RNA was extracted from cell supernatants at 3 d.p.i. and from mouse brain tissue at 7 d.p.i. for RT-qPCR analysis. (**B–F**) The representative Sanger sequencing map and percentage of the corresponding mutations in Aag2 (**B**), BHK-21 (**C**), Vero (**D**), hNPCs (**E**), and neonatal mouse brains (**F**). The C2178 and T2178 are colored in black and blue in the column graph, respectively. (**G**) Cat’s-eye plots of individual relative fitness values in different groups. A relatively replicative fitness greater than one indicates a replication advantage for T2178. The dots represent individual cell lines (*n* = 6) or neonatal mice (*n* = 10). *P* values were calculated for the group coefficient for each linear regression model.

For making each competition internally controlled and eliminating host-to-host variation, competitive experiments were then performed to assay the *in vitro* and *in vivo* fitness of recombinant ZIKV as previously described (21, 26) (Fig. 4A). The nucleotide at position 2178 served as a genetic marker, with cytosine (C) indicating the WT and thymine (T) representing the H401Y substitution. In Aag2 cells, the T allele frequency remained stable at approximately 35% from day 0 to day 3 (Fig. 4B), which aligns with the mutation stability observed in serial passaging experiments (Fig. 1B). Intriguingly, in mammalian cell lines, the T allele proportion increased from approximately 40% to over 60%, increasing the T:C ratio from approximately 0.72 to more than 2.34 (Fig. 4C through E). Notably, the competitive advantage of H401Y was most pronounced in hNPCs, where the T allele represented an overwhelming 82.7% of the population, dramatically increasing the T:C ratio from 0.72 to 8.71 (Fig. 4E). Similarly, in the brains of neonatal mice, the T allele proportion increased from 39.3% on day 0 to 75.6% by day 7, resulting in a T:C ratio increase from 0.72 to 3.55 (Fig. 4F). As shown in Fig. 4G, a relative replicative fitness greater than one indicates a replication advantage for T2178 (21, 26). The relative replicative fitness value of T2178-carrying ZIKV in Aag2 cells was calculated to be 0.97, whereas significantly higher fitness values were detected in Vero, BHK-21, hNPC cells, and neonatal mice, indicating a consistent advantage over C2178-carrying ZIKV in mammals. These results clearly show that the H401Y mutant virus exhibits marked competitive fitness in mammal hosts in contrast to its stability in mosquitoes.

### The H401Y mutant maintains its virulence phenotype in mice after propagation in mosquitoes

**Fig 5:**
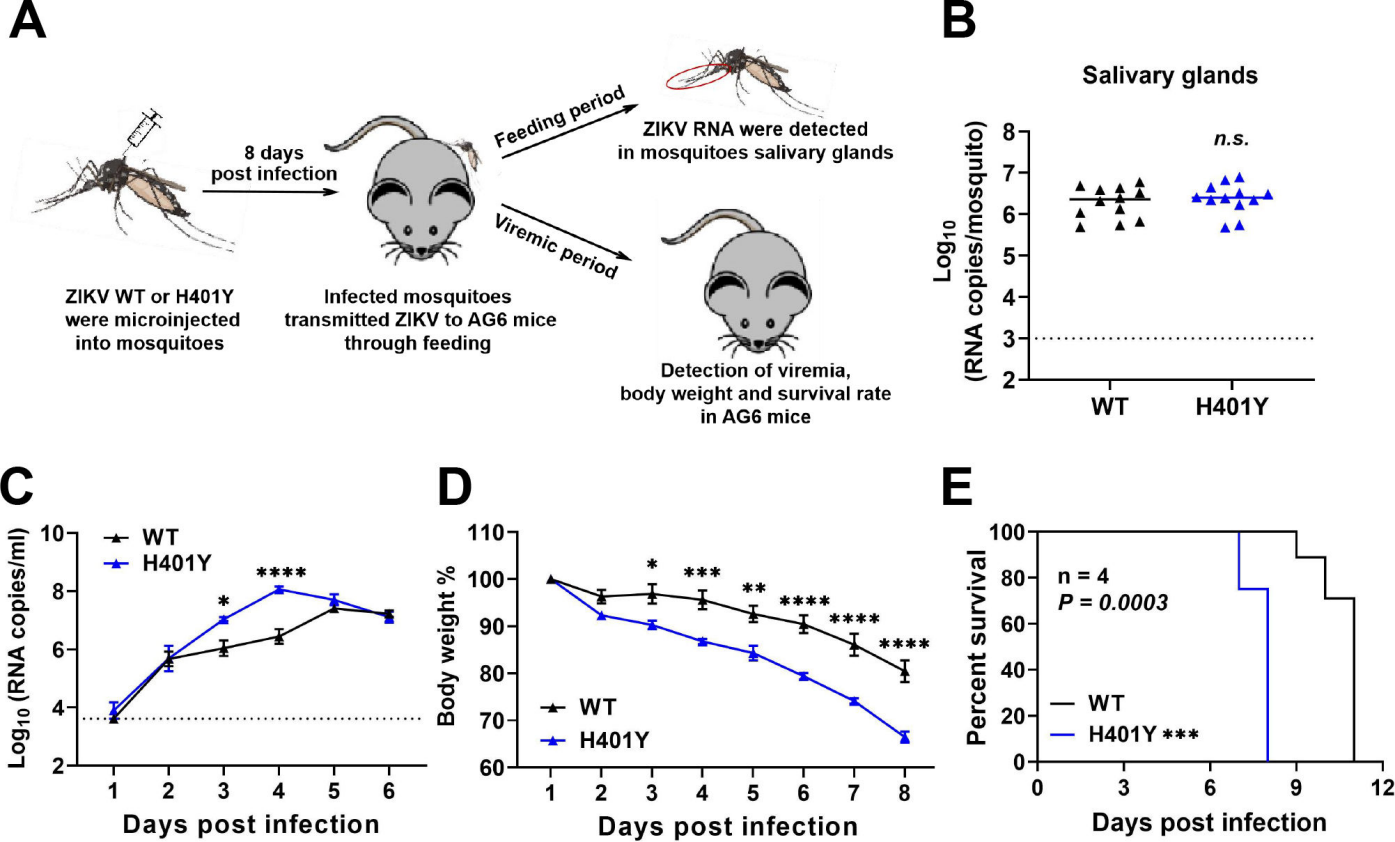
Characterization of the H401Y substitution in the mosquito-mouse transmission cycle. (**A**) Schematic representation of the study design. (**B**) Detection of the ZIKV viral load in the mosquito salivary gland. (**C**) Detection of ZIKV viremia by RT-qPCR (*n* = 4 mice per group). (**D and E**) Comparison of the body weights (**D**) and survival rates (**E**) of infected mice (*n* = 4). (**B**) *P* values were determined using unpaired *t* test. The data are shown as means ± SEM. (**C and D**) The data are shown as means ± SEM. The *P* values were determined by two-way ANOVA (*P < 0.05; **P < 0.01; ***P < 0.001; ****P < 0.0001). (**E**) The survival rate was calculated via Kaplan-Meier survival curves, and multiple comparisons were performed via Log-rank test (***P < 0.001). (**B and C**) The dotted line represents the detection threshold.

Furthermore, the transmission capability of the H401Y virus was evaluated using a mosquito-mouse cycle model (15, 27). As depicted in Fig. 5A, female *Ae. aegypti* mosquitoes were intrathoracically microinjected with equal titers of WT and the H401Y mutant viruses. Approximately 8 days after viral inoculation, three infected mosquitoes were allowed to feed on each AG6 mouse. As expected, the viral load of H401Y in mosquito salivary glands was comparable to that of the WT virus (Fig. 5B). Subsequently, animals exposed to infected mosquitoes developed detectable viremia for each ZIKV, indicating efficient transmission of these strains from infected mosquitoes to mice. However, the H401Y presented a significantly greater viral load in infected mice than did the WT (Fig. 5C). Additionally, mice infected with H401Y through mosquito feeding exhibited accelerated weight loss and a lower survival rate than did those infected with the WT virus (Fig. 5D and E). These results illustrate that the H401Y substitution retains the higher infectivity and virulence phenotype in mice after propagation in mosquitoes.

## DISCUSSION

The replication fidelity of RNA viruses is characteristically low, leading to a prolific accumulation of mutations within their genomic structures. Consequently, these viruses tend to exist as quasispecies. This phenomenon is closely associated with the evolution of viruses, virus-host interactions, and cross-species transmission (28, 29). In this study, the same volume of virus was adopted for passaging to obtain adaptive mutations in mosquito cells. This strategy is also widely used in previous publications (24, 30–32). To keep the MOI constant is the other approach for passaging, which would avoid the drastically different MOIs used in each passage (23, 33–35). Our findings showed that continuous passaging of ZIKV in Aag2 cells resulted in a cumulative increase in mutation sites across the ZIKV genome, but most mutations persisted at frequencies below the 50% threshold (Fig. 1G). In accordance with our findings, Riemersma et al. reported a decrease in genetic similarity across 10 passages in *Ae. aegypti*, with mutation frequencies within the ZIKV genome plateauing below 25% (36). Additionally, in our study, non-synonymous mutations were predominantly observed in the E protein. Sarathy et al. found that single-nucleotide variants in typical ZIKV strains from different lineages were clustered in the prM/M and NS1 genes (37), a pattern that was not observed in our experimental results. This discrepancy may originate from the divergences between laboratory-based passages and natural transmission cycles.

Our experiments revealed that the C2178T mutation (H401Y substitution) obtained from Aag2 cells confers no fitness advantage to wild-type ZIKV in mosquitoes. The C2178T mutation readily emerged during passaging, and the frequency was maintained at approximately 20%–40%. Serial passaging of ZIKV failed to generate a pure ZIKV mutant carrying the C2178T mutation. In this study, the corresponding H401Y mutant was obtained by a reverse genetic system. Notably, genome records from the NCBI database indicate the presence of five complete ZIKV genomes possessing the H401Y substitution (Table 1). Interestingly, the P6-740 strain was subjected to six passages in suckling mouse brains (38), suggesting that serial passaging of ZIKV in mosquito cells or mouse brains likely led to the emergence of the H401Y substitution. Moreover, the other 4 ZIKV strains (15-1144, CTS-178-16p, ARCB116141, and 31N strains) carrying H401Y substitutions have been documented in natural isolates from human and mosquito samples in Asia and the Americas since 2015. Recently, Collins et al. also discovered the H401Y substitution at low frequencies in American ZIKV strains derived from human serum and placenta (37). The factors that drove the emergence of this unique amino acid substitution remain unknown, but the host alternation between mosquitoes and vertebrates in nature might have reduced the risk of further spread of these strains.

**TABLE 1:** ZIKV strains with a tyrosine (Y) residue at position 401 in the E protein from NCBI database

Strain	Isolation source	Country	Collection year
P6-740	*Aedes aegypti* (mosquito)	Malaysia	1966
15-1144	Human serum	Thailand	2015
CTS-178-16p	Blood donor sample	Puerto Rico	2016
ARCB116141	Patient serum	Argentina	2016
31N	*Aedes aegypti* (larvae)	Mexico	2016

The E protein, a crucial determinant of ZIKV virulence, comprises three domains: the central β-barrel domain (DI), the extended dimeric domain (DII), and the immunoglobulin-like domain (DIII) (39). Typically, DIII contains receptor-binding sites in orthoflaviviruses (40) and plays a significant role in facilitating fusion (41). In ZIKV, E-DIII is responsible for receptor binding, comprising strands A–G (42) with the 401-site positioned at the C-terminal end of strand G (396–401). Furthermore, this site is highly conserved among orthoflaviviruses such as West Nile virus (WNV), Japanese encephalitis virus (JEV), and yellow fever virus (YFV) (43), and the role of the H401Y substitution during viral binding, assembly, and immune escape deserves further investigation. In addition, the H401Y mutant virus was rescued in BHK-21 cells, and it is possible that viruses produced in different host cells may introduce some unexpected changes, which may be one of the reasons for their increased infectivity in vertebrate cells.

In summary, we identified a mosquito-accumulated H401Y substitution in the E protein that enhances the fitness, neurovirulence, lethality, and infectivity of ZIKV in mammals. Monitoring and investigating such functionally significant sites are imperative for understanding and shortening outbreaks, as well as for informing disease control strategies.

## MATERIALS AND METHODS

### Cells, viruses, mice, and mosquitoes

Aag2 cells (*Ae. aegypti* cells) were cultured at 28°C in Schneider’s *Drosophila* Medium (Gibco, USA). C6/36 cells (*Aedes albopictus* cells) were maintained at 28°C in RPMI 1640 (Gibco, USA). The mammalian Vero (African green monkey kidney cells) and BHK-21 (baby hamster kidney cells) cell lines were incubated at 37°C in Dulbecco’s modified Eagle medium (DMEM) (Gibco, USA). Each of these media was fortified with 10% fetal bovine serum (FBS), 1% penicillin-streptomycin (P/S), and 1% HEPES, all of which were procured from Gibco (USA). Human neural progenitor cells (hNPCs) were cultured at 37°C in NeuroCult-XF proliferation medium (STEMCELL, Canada), supplemented with heparin solution, epidermal growth factor (EGF), and basic fibroblast growth factor (bFGF).

The ZIKV strain FSS13025 was recovered from an infectious cDNA clone (GenBank accession number KU955593) (44). This clone also served as the backbone for introducing the V153D and H401Y substitutions into the viral envelope protein. A129 and CD-1 mice were acquired from Beijing Vitalstar Biotechnology Corporation, and AG6 mice were acquired from the Institute Pasteur of Shanghai, Chinese Academy of Sciences. *Ae. aegypti* mosquitoes of the Rockefeller strain were reared at Tsinghua University under controlled conditions of 28°C and 80% relative humidity.

### Continuous passage of ZIKV in Aag2 cells

The ZIKV strain FSS13025 underwent 20 continuous passages in Aag2 cells. A day before infection, Aag2 cells were plated on 6-well plates. The following day, the medium was replaced with Schneider’s *Drosophila* medium containing 2% FBS and added to the cells with FSS13025 viral stock at a multiplicity of infection (MOI) of 0.1. The supernatant was collected at 4 d.p.i. and used to infect fresh Aag2 cells; thus, the passage series was continued. Each passage involved mixing 200 µL of the virus from the previous generation with 1,800 µL of medium containing 2% FBS. The supernatants from each passage were stored at −80°C.

### Identification and characterization of mutation sites

Total RNA from passaged viruses was extracted using a QIAamp Viral RNA Mini Kit (Qiagen, USA) and processed for libraries using an Ion Total RNA-Seq Kit V2 (Thermo Fisher, USA). Next-generation sequencing was performed on an Ion GeneStudio S5Plus sequencer (Thermo Fisher, USA). After the removal of adapter sequences and low-quality reads, the remaining clean reads were aligned to the original ZIKV strain FSS13025 (GenBank KU955593) via Bowtie2 (version 2.3.4.3). Single-nucleotide variations (SNVs) were identified with SAMtools (version 1.8) and BCFtools (version 1.10.2). The consensus sequence was then deduced. Mutation sites were visualized via the Integrative Genomics Viewer (IGV, version 2.13.0). Sites exhibiting mutation frequencies of 1% or greater were compiled and graphically represented via GraphPad Prism (version 9.0.0).

### Generation of recombinant Zika virus with specific substitutions

To introduce targeted substitutions at the prM (A94V) and envelope proteins (V153D and H401Y), we employed the Q5 Site-Directed Mutagenesis Kit (NEB, USA) on the pFLZIKV plasmid (44). The corresponding plasmids were transformed into *Escherichia coli* HB101 (TaKaRa, Japan) and subsequently purified using the PureLink HiPure Plasmid Filter Maxiprep Kit (Thermo Fisher, USA). The resulting plasmids were linearized using *Cla*I and transcribed into RNAs using RiboMAX Large Scale RNA Production Systems-T7 (Promega, USA). *In vitro* transcribed viral RNA was puriﬁed using a Purelink RNA Mini Kit (Thermo Fisher, USA). BHK-21 cells were transfected with the *in vitro* transcribed RNA using Lipofectamine 3000 (Thermo Fisher, USA). All virus stocks were further confirmed by sequencing and stored at −80°C until use.

### Plaque-forming assay

A **s**tandard plaque-forming assay of ZIKV was performed in BHK-21 cells. Cells were seeded in 12-well plates 24 h prior to infection. After the culture medium was removed, serial viral dilutions in DMEM supplemented with 2% FBS (400 µL/well) were added, and the mixture was incubated at 37°C for 1 h. The inoculum was then replaced with DMEM containing 2% FBS and 1% low melting-point agarose (Promega, USA). At 4 d.p.i., the overlay was removed, and cells were fixed with 10% formalin and stained with 0.25% crystal violet. Plaques were counted to calculate the virus titers, which were expressed as plaque-forming units per milliliter (PFU/mL).

### Viral RNA quantification by RT-qPCR

Viral RNA was extracted from culture supernatants, mouse serum, or tissue samples using a magnetic bead-based nucleic acid extraction reagent (Tianlong, China). Reverse transcription quantitative PCR (RT-qPCR) was performed with a One Step PrimeScript RT-PCR Kit (TaKaRa, Japan) on a LightCycler 480 real-time PCR system. The RNA in each sample was quantified by targeting the NS5 gene of ZIKV. The following primers and probes were used (18): probe: 5′-6-FAM/AGCCATGACCGACACCACCCGT/BHQ1-3′; ZIKV-F: GGTCAGCGTCCTCTCTAATAAACG; ZIKV-R: GCACCCTAGTGTCCACTTTTTCC. The determination of viral RNA copy number was performed by referring to a standard curve. Brieﬂy, the pFLZIKV plasmid containing the full-length genomic cDNA clones of ZIKV was subjected to *in vitro* transcription. *In vitro* transcribed viral RNA was puriﬁed and quantiﬁed using spectrophotometry on a Nanodrop 2000. The puriﬁed RNA was serially diluted 10-fold and detected using RT-qPCR. The resultant standard curve was used to determine the viral RNA copy number in the samples.

### Virus growth curve analysis in cell lines

Growth curves for ZIKV were generated for Aag2, C6/36, Vero, BHK-21, and hNPCs. Cells seeded onto 48-well plates were infected at an MOI of 0.1 or 1. The supernatants were collected and subjected to viral RNA quantification by plaque assay. All the experiments were performed in triplicate.

### Indirect immunofluorescence assay

BHK-21 cells were infected with virus at an MOI of 0.1 and fixed with methanol/acetone (7:3) at 24, 48, and 72 h post infection (h.p.i.). The cells were incubated with anti-ZIKV E protein (1:1,000 diluted, BioFront Technologies, China) at 4°C overnight after being washed with PBS three times. Then, the cells were incubated with Alexa Fluor 488 (1:200 diluted, GeneTex, USA) at 37°C for 1 h after being washed with PBS three times. Fluorescent images were acquired via a fluorescence microscope (Zeiss, Germany).

### Competition assays *in vitro* and *in vivo*

For the competition assay, Aag2, Vero, BHK-21, and hNPCs were plated onto 48-well plates the day prior to infection. The cells were concurrently infected with a 1:1 PFU ratio mixture of WT and H401Y mutant ZIKV at an MOI of 0.1 (1 for hNPCs), for 1 h, and after infection, the cells were washed three times with PBS and incubated at 37°C, or 30°C for Aag2 cells. Supernatants were harvested at 3 d.p.i. Additionally, one-day-old CD-1 mice received intracranial injections of 100 PFU of the same WT or H401Y virus mixture, and their brains were collected at 7 d.p.i. Viral RNA was extracted, reverse-transcribed using a one-step RT-PCR Kit (TaKaRa, Japan) and sequenced. The primers used were FSS-F3: GGTTTTGGAAGCCTAGGACTT and FSS-R3: GGGAAATAGATCCATTCTTTGTATTCAG, and the resulting base ratios were analyzed by SnapGene. Relative replicative fitness values for the T2178 strain over the C2178 strain in each sample were analyzed according to *w* = (f0/i0), where i0 is the initial T2178/C2178 ratio and f0 is the final T2178/C2178 ratio after the competition.

### Mouse experiments for neurovirulence

CD-1 mice were obtained at 16 days of gestation, and their pups were utilized for experiments one-day post-delivery. The pups were intracranially injected with 10 PFU of either WT or H401Y ZIKV to evaluate neurovirulence. The mice were monitored for up to 21 days for survival curves (*n* = 9). Serum and brains from similarly injected mice were collected at different time point post infection for viral RNA quantification, histopathological analysis, and immunofluorescence assays.

### Mouse experiments for neuroinvasiveness assessment

A group of 3-week-old male A129 mice were intraperitoneally injected with 10^5^ PFU of either WT or H401Y virus to assess neuroinvasiveness. These mice were observed for up to 14 days for survival (*n* = 9). Serum samples (*n* = 5) were collected at 1 and 3 d.p.i., and brain tissue samples (*n* = 5) were collected at 7 d.p.i. for viral RNA quantification.

### Histopathological and immunofluorescence analysis

Two brain tissues were randomly collected at 9 d.p.i. and made into slices of coronal and sagittal planes, respectively. For histopathological examination, neonatal brain tissues were fixed in 4% neutral buffered formaldehyde for 24 h, embedded in paraffin, and sectioned into 4 µm slices. The section was stained with hematoxylin and eosin (H&E) and inspected under a light microscope. For immunofluorescence staining, the section was treated with 0.5% Triton X-100 in PBS and 0.01 M sodium citrate. After the samples were blocked with 10% bovine serum albumin, they were incubated with the following primary antibodies: mouse anti-ZIKV envelope protein antibody (1:500 dilution; BioFront Technologies, China) or rabbit anti-NeuN overnight at 4°C (1:1,000 dilution; CST, USA). Following PBS washes, the sections were incubated with an Alexa Fluor 488-conjugated anti-mouse antibody (1:300 dilution; GeneTex, USA) or an Alexa Fluor 575-conjugated anti-rabbit antibody (1:300 dilution; Thermo Fisher, USA) for 2 h at 37°C. Nuclei were counterstained with DAPI (Abcam, UK). Confocal laser scanning microscopy was employed for image acquisition.

### Mosquito infectivity assay

Groups of female *Ae. aegypti* mosquitoes (*n* = 9–12) were inoculated intrathoracically as previously described (45). Briefly, female mosquitoes were first immobilized by placing them on a cold tray and then microinjected with a precise volume of ZIKV at a concentration of 50 PFU/mL directly into their thoracic cavity. We then measured the ZIKV load in the whole mosquitoes at 3 and 6 d.p.i. via RT-qPCR.

### Mosquito-mouse transmission experiments

Female *Ae. aegypti* mosquitoes were inoculated intrathoracically with ZIKV (50 PFU/mL). Following inoculation, the mosquitoes were placed in cups covered with netting and were fasted for 24 h before they were allowed to feed. Groups of three mosquitoes were permitted to feed on a single 6-week-old AG6 mouse at 8 d.p.i. For this process, the AG6 mice were anesthetized and positioned atop the mosquito-containing container. The mosquitoes were then allowed a 20-min feeding period in darkness. After feeding, the mosquitoes were anesthetized via ice, and their engorged salivary glands were surgically removed for viral load analysis via RT-qPCR. From 1 to 6 d.p.i., blood samples were collected from the infected mice to determine the viral load via RT-qPCR. Additionally, changes in the body weights and survival rates of these mice were meticulously recorded until their death.

### Statistical analysis

The data were analyzed using GraphPad Prism software (version 9.0.0). Statistical comparisons of the results were made using unpaired *t* test, one-way ANOVA or two-way ANOVA with Tukey’s multiple comparisons test. Log10 conversion of viral RNA copies or PFU was performed, followed by corresponding statistical analysis. The results were shown as the mean ± standard deviation (SD) or as specified in the figure legends accompanying the findings. Log-rank tests were performed for survival analysis. *P* values are denoted as follows (n.s., not significant, *P < 0.05, **P < 0.01, ***P < 0.001, ****P < 0.0001).

For the virus competition assay, statistical methods established by Plante et al. were used (26). In brief, to model f0/i0, log10 is transformed to an improved approximation of normality and modeled by analysis of variance in relation to the group, adjusting by experiment to control for clustering within the experiment. Specifically, the model was of the form Log10_CountT1overCount T0 ~Experiment + Group. The fitness ratios between the two groups [the model’s estimate of *w* = (f0/i0)] were assessed per the coefficient of the model’s Group term, which was transformed to the original scale as a 10^coefficient. Statistical analyses were performed using R statistical software (R Core Team, 2019, version 3.6.1). In all the statistical tests, two-sided alpha = 0.05 was used. Cat’s-eye plots (46), which illustrate the normal distribution of the model-adjusted means, were produced using the catseyes package (47).

## Data Availability

The sequencing data have been uploaded to the SRA database under accession number PRJNA1096993. Five biosamples, P0, P5, P10, P15, and P20, are included.
